# Evaluating a Two-Cycle Quality Improvement Project Using Multi-Cohort Thematic Feedback Analysis of Year 4 Undergraduate Psychiatry Placements at a National Health Service (NHS) Trust in Northwest England

**DOI:** 10.1192/bjo.2026.11444

**Published:** 2026-06-30

**Authors:** Mohamed Nemary, Nishanth Babu Mathew, Emily Wing

**Affiliations:** Mersey Care NHS Foundation Trust, Liverpool, United Kingdom

## Abstract

**Aims:**

To evaluate the effectiveness of interventions implemented following a first-cycle quality improvement (QI) project (2023/2024) aimed at enhancing the educational experience of Year 4 medical students during psychiatry placements, and to identify persistent or emerging themes requiring further intervention during the second cycle (2024/2025).

**Methods:**

Qualitative feedback was collected routinely from Year 4 medical students following their 4-week psychiatry placements across a large NHS Trust in Northwest England. Free-text comments (187) were thematically analysed across multiple cohorts (7). A frequency threshold of three to four mentions was used to define a feedback theme, with issues appearing fewer than three times excluded from thematic categorisation. Themes were compared with those identified in the first QI cycle to evaluate progress, sustained improvements, unresolved issues, and areas of regression. Frequency counts were used to quantify the prominence of each theme.

**Results:**

Sustained improvements were observed following first-cycle interventions, including administrative organisation and communication, induction and wellbeing support, and improved access to portfolio opportunities. However, multiple themes persisted or showed limited improvement. Timetabling and clinical exposure remained a concern (7 mentions), with students reporting ward-heavy schedules, limited community experience, and fragmented Fridays. Dissatisfaction with online Case-Based Learning (CBL) continueddespite increased face-to-face delivery (14 mentions). Site induction issues persisted (5 mentions), with students reporting inadequate or absent inductions. Access to senior clinicians for supervision and Case-Based Discussions (CBDs) remained variable (4 mentions). Safety and logistical issues, including shortages of keys and alarms, were also noted (4 mentions), alongside reports of excessive travel and midday site changes (3 mentions). Areas of regression were identified in Child and Adolescent Mental Health Services (CAMHS) placement organisation (11 mentions), where students described disorganisation, non-attendance of patients, and limited clinic exposure. Concernsabout Balint group timing persisted (4 mentions), contributing to fragmented Fridays, and some students needed to rearrange schedules to meet portfolio requirements (3 mentions).

**Conclusion:**

The secondcycle QI evaluation demonstrates meaningful progress in administrative organisation, induction quality, and portfolio support. However, persistent challenges remain in timetabling, CAMHS organisation, online teaching formats, and access to senior supervision. These findings highlight the need for continued Plan–Do–Study–Act (PDSA)driven interventions, strengthened site-level induction processes, improved scheduling, and closer collaboration with CAMHS teams to ensure a consistently high-quality undergraduate learning experience.

